# Evaluating the Efficacy and Safety of Aspirin for Primary Cardiovascular Prevention in Asian Patients with Type 2 Diabetes: A Population-Based and Propensity Score-Matched Study

**DOI:** 10.3390/diagnostics14121211

**Published:** 2024-06-07

**Authors:** Kai-Wei Chang, Jing-Yang Huang, Shun-Fa Yang, Kwo-Chang Ueng

**Affiliations:** 1 Division of Cardiology, Department of Internal Medicine, Chung Shan Medical University Hospital, Taichung 402, Taiwan; 2 School of Medicine, Chung Shan Medical University, Taichung 402, Taiwan; 3 Center for Health Data Science, Chung Shan Medical University Hospital, Taichung 402, Taiwan; 4 Institute of Medicine, Chung Shan Medical University, Taichung 402, Taiwan; 5 Department of Medical Research, Chung Shan Medical University Hospital, Taichung 402, Taiwan

**Keywords:** aspirin, cardiovascular prevention, type 2 diabetes, propensity score matched

## Abstract

The risk of developing cardiovascular disease is significantly higher for individuals with diabetes compared to those without. Aspirin has been widely used for primary prevention in diabetic patients. However, evidence is limited in the Asian population. We aimed to compare the effectiveness and safety of aspirin versus placebo for primary cardiovascular prevention in the Asian population with type 2 diabetes. In this study, we performed propensity score matching with non-aspirin users from January 2006 to December 2015 (*n* = 37,095 in each group after matching, PSM). We analyzed the incidence risk of all-cause mortality, composite cardiovascular events, and hospitalized major bleeding. The propensity score-matched (PSM) cohort of patients who received aspirin within one year of diabetes diagnosis was compared with the non-aspirin diabetic (DM) cohort. Baseline characteristics were balanced between the two groups. The median follow-up duration was 78 months. Aspirin users exhibited a slightly but significantly lower rate of all-cause mortality (HR: 0.92; 95% CI: 0.87 to 0.96). However, they also had a significantly higher composite cardiovascular risk (HR: 1.34; 95% CI: 1.28–1.40), including non-fatal acute myocardial infarction (HR: 1.33; 95% CI: 1.18 to 1.50), non-fatal ischemic stroke (HR: 1.38; 95% CI: 1.30 to 1.45), heart failure (HR: 1.18; 95% CI: 1.09 to 1.27), and coronary revascularization (HR: 1.94; 95% CI: 1.73 to 2.17). Aspirin users also faced a significantly higher risk of hospitalized major bleeding (HR: 1.08; 95% CI: 1.03 to 1.14). The presence of one or more additional risk factors did not influence the effectiveness and safety outcomes of aspirin, according to stratified analysis. In conclusion, in this real-world Asian diabetic population, aspirin was associated with a significantly lower mortality risk but also with higher risks of cardiovascular events and hospitalized bleeding. Aspirin may not play a role in the primary prevention of cardiovascular disease in such patients, regardless of additional risk factors.

## 1. Introduction

Despite the lack of robust evidence, the 2021 American Diabetes Association (ADA) “Standards of Medical Care in Diabetes” suggested that the low-dose aspirin therapy may be considered a primary prevention strategy in high-risk diabetic patients [1]. This question may be more important for Asians, since the clinical guidelines of aspirin use in the primary prevention of cardiovascular events in Western countries may not be appropriately applied to Asian populations due to different disease preference in this population. Recently, a large, randomized control trial (ASCEND trial) compared aspirin and placebo in a diabetic population who had no evidence of cardiovascular disease (CVD) [2]. The aspirin therapy prevented serious vascular events compared with the placebo, but it also significantly increased the risk of major hemorrhage. However, the ASCEND trial enrolled a very limited number of Asian patients (<1%), and it might not be possible to extrapolate the results to all races.

Limited previous aspirin primary prevention trials in diabetic patients were mainly conducted in Western countries, except the JPAD trial [3], which included only 2539 diabetic patients. Moreover, East Asian patients tend to have more bleeding events and less thromboembolic events when compared to Caucasian patients. Whether the use of low-dose aspirin could prevent primary CVD remains unclear in patients with diabetes in Asia. Therefore, we conducted the largest Asian cohort study to evaluate the effectiveness and safety of low-dose aspirin in the primary prevention of CVD in diabetic patients based on the National Health Insurance Research Database (NHIRD) of Taiwan.

## 2. Materials and Methods

### 2.1. Data Source

The data for this study were sourced from Taiwan’s NHIRD, one of the largest administrative healthcare databases globally. It covers healthcare data for more than 99% of the population (approximately 23 million residents) in Taiwan since 2010. Under Taiwan’s universal National Health Insurance (NHI) scheme, all medical claims are mandatorily submitted to the Bureau of National Health Insurance (BNHI) for validation and reimbursement. The NHIRD compiles beneficiaries’ registration files, which include demographics, all types of medical visits, procedure codes, prescription codes, and diagnostic codes according to the International Classification of Diseases, 9th Revision, Clinical Modification (ICD-9-CM). The Institutional Review Board of Chung Shan Medical University in Taiwan approved this study (IRB number CS-16183) and waived the requirement for informed consent, as the data were anonymized and de-identified prior to analysis.

### 2.2. Study Population

The study utilized a retrospective cohort and new user design to evaluate the effectiveness and safety of aspirin for the primary prevention of cardiovascular events in patients with diabetes. We excluded patients with a pre-existing diagnosis of diabetes mellitus (DM) who had been seen for DM before 2006 (*n* = 970,763) and those who had taken aspirin prior to 2006 (*n* = 12,167). Consequently, 1,481,618 patients newly diagnosed with DM from 2006 to 2015 were included. In this new user design, patients who received any oral antiplatelet other than aspirin or anticoagulant therapy before the index date were also excluded. The index date was defined as 365 days post-aspirin initiation.

### 2.3. Long-Term Low-Dose Aspirin Exposure for Primary Prevention

The key independent variable was the initiation of daily low-dose aspirin therapy, defined as 75 to 100 mg per day, in subjects with diabetes. Aspirin exposure was specified as diabetic patients receiving low-dose aspirin treatment for at least 90 days within the first year from the initial prescription. A total of 225,701 patients were identified as aspirin users. Considering the challenges of estimating short-term effectiveness in an observational study design, we set the index date to 365 days after the initiation of aspirin therapy. To focus on primary prevention, we excluded patients who had pre-existing conditions such as coronary artery disease (CAD), peripheral arterial occlusive disease (PAOD), heart failure, or stroke before the index date. We analyzed only the events occurring one year after the initiation of aspirin therapy to minimize the potential for reverse causality. Additionally, we excluded patients who had received any other oral antiplatelet such as clopidogrel or any antithrombotic therapy prior to the index date. Ultimately, 41,587 patients constituted the low-dose aspirin cohort.

### 2.4. Patient Characteristics and Selection of Study Control

Disease diagnoses were defined using ICD-9-CM codes, and information on prescriptions (including drug ID, prescription date, Anatomical Therapeutic Chemical code, drug components, dosage, and prescription day) from outpatient visits and hospitalizations was retrieved for the period 2006 to 2016. To control for potential confounding biases in the risk assessment of mortality and non-fatal major cardiovascular events, we also identified comorbidities (including hypertension, chronic liver diseases, malignancy, hyperlipidemia, chronic kidney diseases, peptic ulcer, gout, and dementia) and the use of co-medications (including alpha-blockers, beta-blockers, calcium channel blockers, ACE inhibitors/ARBs, diuretics, statins, fibrates, NSAIDs, corticosteroids, proton pump inhibitors, biguanides, sulfonylureas, alpha-glucosidase inhibitors, thiazolidinediones, DPP-4 inhibitors, and insulin).

Initial matching was conducted by age, sex, and year of DM diagnosis. Each aspirin user was individually matched with two controls. Throughout this process, we ensured all controls met the exclusion criteria at the matched index date. After age–sex matching, index dates and baseline characteristics were established for each participant. Propensity score matching was subsequently performed to further balance the baseline characteristics. Follow-up was conducted from the index date until the occurrence of study events, death, or the end of the study period (December 2016).

### 2.5. Study Events

To ensure diagnostic accuracy, DM was defined only when confirmed by at least two outpatient claims or one inpatient claim. This diagnostic criterion for DM in the NHIRD has been validated previously [4]. Follow-up time was calculated from enrollment to the occurrence of the first major vascular event, death, or until December 31, 2016, whichever came first. We identified clinical outcomes to assess the effectiveness and safety of aspirin. The primary endpoints were defined as all-cause mortality and composite cardiovascular events, which included hospitalizations due to non-fatal acute myocardial infarction (AMI, ICD-9: 410–412), non-fatal stroke (ICD-9: 433–438), heart failure (ICD-9: 428), coronary revascularization (either percutaneous coronary intervention or coronary artery bypass surgery), and all causes of death, whichever occurred first. Safety endpoints included all hospitalized bleeding events, such as gastrointestinal bleeding (ICD-9: 578), intracranial bleeding (ICD-9: 430–432), hematuria (ICD-9: 599.7), and airway bleeding (ICD-9: 786.3).

### 2.6. Subgroup Analysis

Subgroup analyses were conducted based on age, sex, hypertension, hyperlipidemia, and chronic kidney disease (CKD). For the primary composite endpoints, we also assessed the impact of statin and renin–angiotensin system (RAS) inhibitor use, including the use of ACE inhibitors/ARBs and statins, on the effectiveness of the treatment. Patients were categorized by age into groups younger than 75 and those 75 or older. For conditions such as hypertension, hyperlipidemia, and CKD, patients were classified according to the presence or absence of these diseases. The statistical significance of the interactions between treatment and specific subgroups was evaluated in each subgroup analysis

### 2.7. Statistical Analysis

Data were presented as frequencies (proportions) for categorical variables. Propensity score matching (PSM) used the greedy, nearest-neighbor method without replacement and a caliper of 0.01 of the propensity score to ensure precise matching. The balance of baseline characteristics between aspirin users and non-users was assessed using the absolute standardized difference (ASD), with an ASD of <0.01 indicating negligible differences between the cohorts for each covariate. Incidence rates (per 1000 person-years) and their 95% confidence intervals for study events were calculated using Poisson regression. Multivariable Cox proportional hazards regression, adjusted for potential confounders including age, gender, duration of diabetes, and baseline use of nonsteroidal anti-inflammatory drugs, corticosteroids, proton pump inhibitors, antihypertensives, antidiabetics, and lipid-lowering drugs, estimated the risk of study events. The competing hazard ratio considered all causes of death as competing events. The cumulative risk of study events for aspirin users compared to non-users was analyzed using the Kaplan–Meier method and log-rank test for hypothesis testing. Statistical significance was set at *p* < 0.05, and all tests were two-tailed. All analyses were performed using SAS 9.4 (SAS Institute Inc., Cary, NC, USA).

## 3. Results

### 3.1. Baseline Characteristics

**Figure 1:**
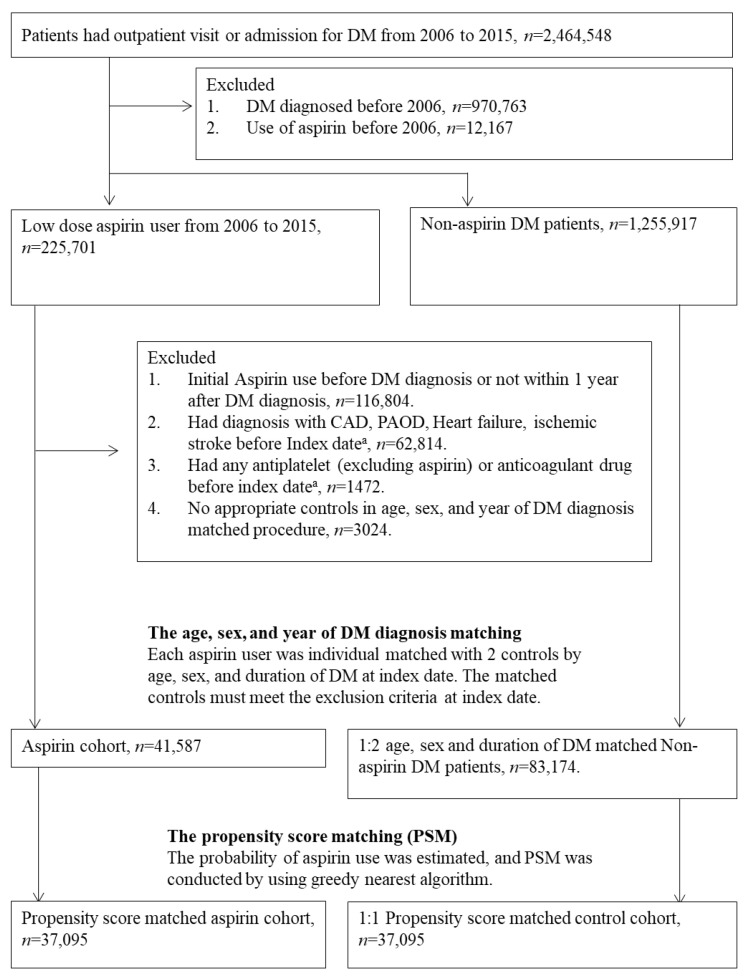
Flow chart for selection of study groups^. a^ Index date was defined as the date one year after first aspirin therapy. CAD = coronary artery disease; PAOD = peripheral arterial occlusive disease.

A total of 41,587 patients, all at least 20 years old with type 2 diabetes who were newly administered aspirin, were included in the study. We selected 83,174 type 2 DM patients not on low-dose aspirin as the control group, matching them in a 1:2 ratio by age, sex, and year of DM diagnosis. Before propensity score matching, aspirin users exhibited a more adverse cardio-metabolic risk profile and higher usage of cardiovascular drugs, anti-diabetic drugs, and statins compared to non-users (Table 1). After propensity score weighting, the characteristics of aspirin users and non-users were well balanced (all ASDs < 0.100), as shown in Table 1. Post-matching, the study included 37,095 aspirin users and an equal number of non-users (Figure 1). The mean age of the matched population was 57.98 years, with males comprising 56.77% of the aspirin cohort, showing homogeneity with the non-aspirin cohort (ASD = 0.008). The median follow-up duration was 78 months for aspirin users and 79 months for non-users.

### 3.2. Risk of All-Cause Mortality and Clinical Cardiovascular Outcomes

Over an average follow-up of 6.5 years, the risk of all-cause mortality was 14.49 events per 1000 person-years among aspirin users, significantly lower than 15.72 events per 1000 person-years in propensity score-matched non-users (aHR: 0.92; 95% CI: 0.87–0.96). However, aspirin use was associated with worse cardiovascular outcomes compared to non-use after PSM (Table 2). The primary composite endpoints (non-fatal AMI, heart failure, non-fatal ischemic stroke, and coronary revascularization) occurred more frequently in aspirin users (20.45 per 1000 person-years) compared to non-users (15.67 per 1000 person-years). Specifically, the incidence rates for non-fatal AMI were 2.58 per 1000 person-years in aspirin users versus 1.94 in non-users (aHR: 1.33; 95% CI: 1.18–1.50). Non-fatal ischemic stroke occurred at a rate of 12.54 per 1000 person-years among aspirin users, compared to 9.34 per 1000 person-years among non-users (aHR: 1.38; 95% CI: 1.30–1.45). Additionally, the incidence of heart failure was higher in aspirin users (6.55 per 1000 person-years) than in non-users (5.65 per 1000 person-years; aHR: 1.18; 95% CI: 1.09–1.27), and the incidence of undergoing coronary revascularization was also higher among aspirin users (3.66 vs. 1.90 per 1000 person-years; aHR: 1.94; 95% CI: 1.73–2.17).

**Figure 2:**
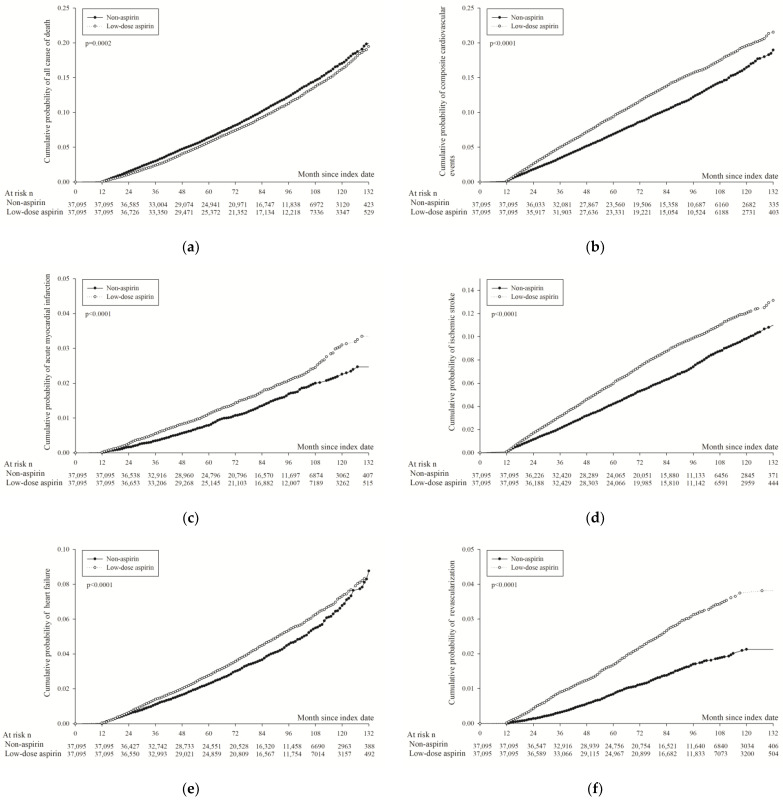
Kaplan–Meier curves of cumulative probability of efficiency of study events in propensity score-matched aspirin and control group; *p* value was obtained using Fine and Gray method. (**a**) All-cause mortality. (**b**) Composite cardiovascular events. (**c**) Acute myocardial infarction. (**d**) Ischemic stroke. (**e**) Heart failure. (**f**) Coronary revascularization.

The Kaplan–Meier curves, illustrating the cumulative probability of effectiveness for study events, are displayed in Figure 2. The curves demonstrate that the cumulative probability of all-cause mortality in aspirin users consistently remained lower than in non-users throughout the follow-up period (*p* < 0.001; Figure 2a). Conversely, the cumulative probability of composite cardiovascular events was higher among aspirin users from the onset of follow-up (*p* < 0.001; Figure 2b). Similar trends were observed for other primary endpoints, including non-fatal AMI (*p* < 0.001; Figure 2c), non-fatal ischemic stroke (*p* < 0.001; Figure 2d), heart failure (*p* < 0.001; Figure 2e), and coronary revascularization (*p* < 0.001; Figure 2f).

### 3.3. Safety Endpoint of Hospitalized Major Bleeding

**Figure 3:**
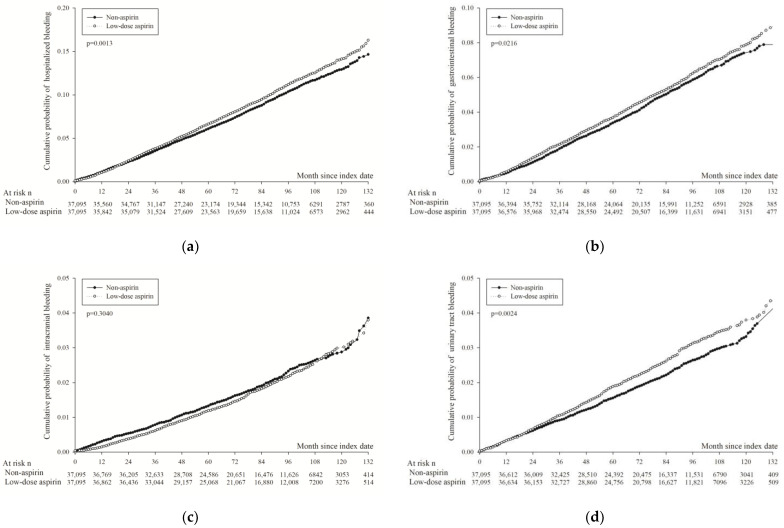
Kaplan–Meier curves of cumulative probability of hospitalized bleeding in propensity score-matched groups; *p* value was obtained using Fine and Gray method. (**a**) Hospitalized bleeding. (**b**) Gastrointestinal bleeding. (**c**) Intracranial bleeding. (**d**) Hematuria.

Table 2 indicates that low-dose aspirin was associated with an 8% higher risk of major bleeding leading to hospital admission compared to non-users (aHR: 1.08; 95% CI: 1.03–1.14). The incidence of the first hospitalized gastrointestinal bleeding was 9.69 per 1000 person-years among aspirin users, slightly higher than 9.33 in non-users (aHR: 1.08; 95% CI: 1.01–1.16). The incidence of intracranial bleeding was similar between groups: 3.48 per 1000 person-years for aspirin users and 3.81 for non-users (aHR: 0.09; 95% CI: 0.85 to 1.05). However, the incidence of urinary tract bleeding was higher in aspirin users, with 5.31 per 1000 person-years compared to 4.91 in non-users (aHR: 1.10; 95% CI: 1.05–1.28). Figure 3 displays the Kaplan–Meier curves for the cumulative probability of safety-related study events. As expected, aspirin users demonstrated a higher cumulative probability of bleeding events, including hospitalized bleeding (*p* < 0.001; Figure 3a), gastrointestinal bleeding (*p* < 0.001; Figure 3b), and urinary tract bleeding (*p* < 0.001; Figure 3d). No significant difference was observed in the incidence of intracranial bleeding between the groups (*p* = 0.304; Figure 3c).

### 3.4. Subgroup Analysis

**Figure 4:**
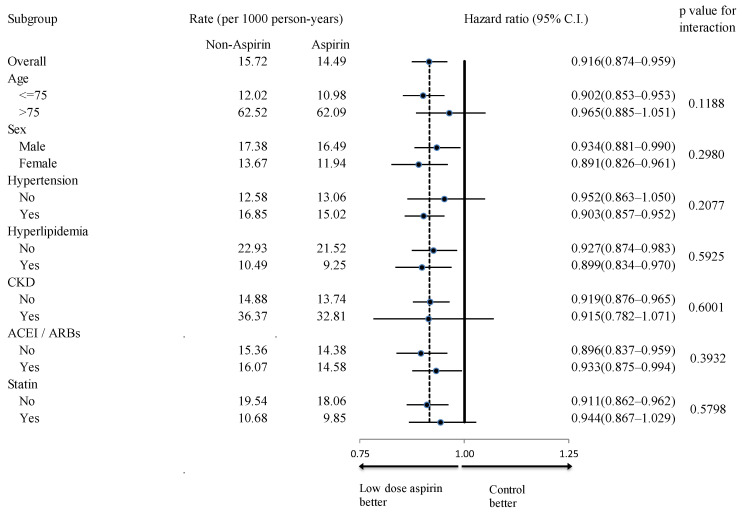
Subgroup analysis of the effect of aspirin use (vs. non-users) on the occurrence of all-cause mortality. CKD, chronic kidney disease; ACEI, angiotensin-converting enzyme inhibitor; ARB, angiotensin receptor blockers.

**Figure 5:**
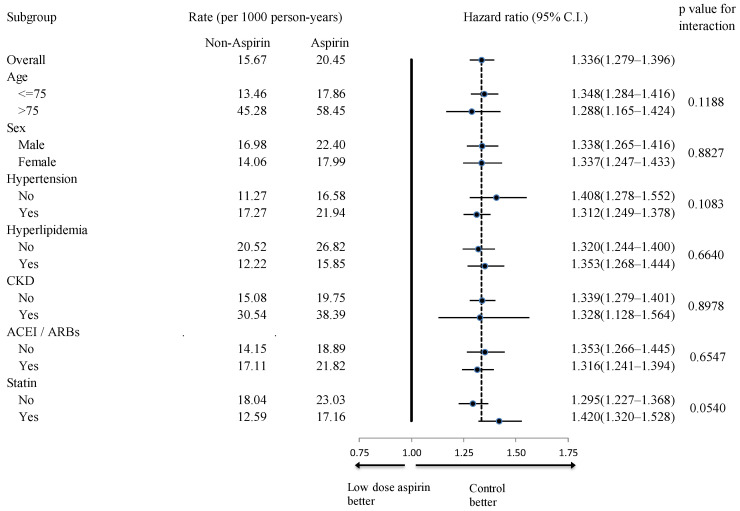
Subgroup analysis of the effect of aspirin use (vs. non-users) on the occurrence of primary composite endpoints. CKD, chronic kidney disease; ACEI, angiotensin-converting enzyme inhibitor; ARB, angiotensin receptor blockers.

**Figure 6:**
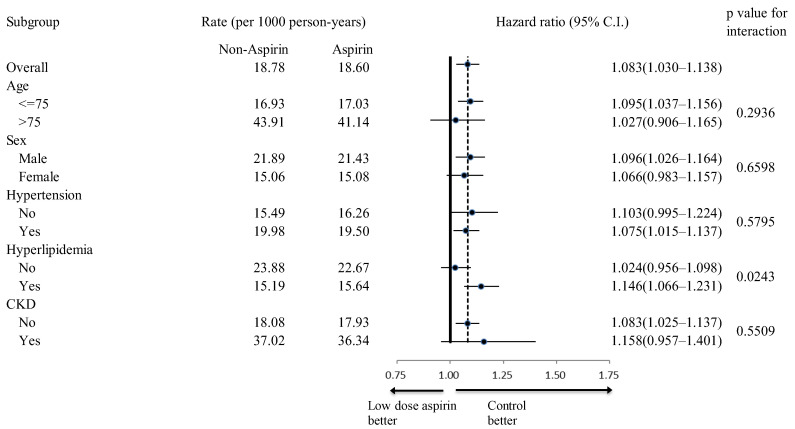
Subgroup analysis of the effect of aspirin use (vs. non-users) on the occurrence of hospitalized bleeding. CKD, chronic kidney disease.

In subgroup analyses, the aspirin group exhibited fewer all-cause mortality events across all examined subgroups (Figure 4). Additionally, aspirin users showed a higher incidence rate of primary composite endpoints in all subgroups, regardless of age, sex, hypertension history, hyperlipidemia history, chronic kidney disease (CKD) history, concomitant ACEI/ARB use, and concomitant statin use (Figure 5). While aspirin users generally faced a higher risk of all hospitalized bleeding events across most subgroups, this increased risk was not observed in the subgroup of patients without hyperlipidemia (*p* for interaction = 0.0243) (Figure 6).

In a stratified analysis by the number of risk factors (Table 3), aspirin did not provide additional benefits for all-cause mortality in patients with a higher number of risk factors (*p* for interaction = 0.56). This trend was consistent for composite cardiovascular events (*p* for interaction = 0.35), including non-fatal AMI (*p* for interaction = 0.60) and heart failure (*p* for interaction = 0.56). Notably, among those with fewer risk factors, aspirin users showed a higher adjusted hazard ratio (aHR) compared to non-users for non-fatal ischemic stroke (*p* for interaction = 0.028), indicating potential benefits in patients with more risk factors. In contrast, for all hospitalized bleeding events, aspirin users had a higher aHR irrespective of the number of risk factors (*p* for interaction = 0.54).

## 4. Discussion

This was the biggest, real-world, nationwide population-based study to investigate the effectiveness and safety of aspirin in the primary prevention of cardiovascular disease with a focus on Asian diabetic patients. No study has reported the effectiveness and safety of aspirin in primary prevention in a population-based cohort. In this Asian population consisting of 1,481,618 diabetic patients without prior CVD, 159,391 subjects (~17.97%) were treated with low-dose aspirin as primary prevention therapy. Our study showed that aspirin was associated with a lower risk of all-cause mortality (aHR: 0.92; 95% CI: 0.85 to 0.96). Unexpectedly, in this propensity score-matched study design, aspirin was associated with an increased risk of composite cardiovascular events (primary composite endpoints) (aHR: 1.34; 95% CI: 1.28 to 1.40). Separately, the risks of acute myocardial infarction, ischemic stroke, heart failure, and coronary revascularization were all increased in aspirin users. Aspirin was also associated with an increased risk of all hospitalized bleeding (aHR: 1.08; 95% CI: 1.03 to 1.14), gastrointestinal bleeding, and hematuria.

In the general population, aspirin therapy as a primary prevention of CVD is controversial. Previous meta-analysis studies revealed that the benefits of cardiovascular protection are offset by clinically important bleeding events [5,6]. Recently, the ASPREE study compared low-dose aspirin vs. placebo as a primary prevention strategy in the healthy elderly (≥70 years old). The results showed a significantly higher risk of major bleeding in the aspirin treatment group compared with the placebo group (hazard ratio: 1.38; 95% CI: 1.18 to 1.62) without a significant reduction in the risk of CVD (hazard ratio: 0.95; CI: 0.83 to 1.08) [7].

Diabetes mellitus is no doubt the major risk factor of CVD [8,9]. Since aspirin therapy has strong evidence in secondary prevention [5,10], it seems reasonable to use aspirin in diabetic patients to prevent CVD. However, previous studies showed conflicting results [11,12,13]. In the ASCEND trial, which compared aspirin and placebo in a diabetic population who had no evidence of CVD [2], there were significantly lower serious vascular events in the aspirin group than in the placebo group (rate ratio: 0.88; CI: 0.79 to 0.97). In the exploratory analysis, the benefit of aspirin was mainly seen in the first 5 years (<3 years, rate ratio: 0.74; CI: 0.63 to 0.89; 3 to <5 years, rate ratio: 0.85; CI: 0.69 to 1.04; ≧5 to <7 years, rate ratio: 1.04; CI: 0.84 to 1.28). In our study, however, from the KM plot of composite cardiovascular events, we did not find that aspirin intervention reduced early CVD risk. The hazard ratio of composite cardiovascular events did not change over time, and the cumulative incidence probability was higher in aspirin users since follow-up (*p* < 0.001; Figure 2b).

Another RCT conducted in Japan (the JPAD trial) [3] also compared low-dose aspirin and placebo as a primary prevention in diabetic patients. The results showed that aspirin did not reduce the risk of all atherosclerotic events (hazard ratio: 0.80; 95% CI: 0.58 to 1.10). However, aspirin did reduce the risk of the combined endpoint of fetal coronary events and fetal cerebrovascular events (HR: 0.10; 95% CI: 0.01 to 0.79). The 10-year follow-up data of the JPAD trial showed the consistent result that the aspirin did not reduce cardiovascular events compared with the placebo in the per-protocol cohort (hazard ratio: 1.14; 95% CI: 0.91 to 1.42) [14].

The ASCEND trial included a total of 15,480 participants, and the majority of participants were white (96.5%). Only 1% of participants were South Asian and 1% were African or Caribbean. The JPAD trial included a total of 2539 Japanese diabetic patients. To the best of our knowledge, the present study is the largest cohort of Asian patients with diabetes who were prescribed aspirin as the primary prevention of cardiovascular disease. Compared with the ASCEND trial, this study had younger participants (57.98 years old versus 63.2 years old), less males (56.3% versus 62.6%), more hypertension (74.64% versus 61.6%), and shorter duration of diabetes mellitus (<1 year versus 7 years). In this study, 58% of patients had a diagnosis of hyperlipidemia, and 43% of patients received statin therapy, which was less than in the ASCEND trial (75% statin use). Compared with the JPAD trial, this study also had younger participants (57.98 years old versus 64 years old in JPAD), more males (56.2% versus 53%), more hypertension (74.6% versus 57%), and more hyperlipidemia (57.8% versus 52%). Only 26% of patients in the JPAD trial received statin therapy. Another significant difference between this study, the ASCEND trial, and the JPAD trial was the duration of diabetes before aspirin therapy. In this study, we included patients who received aspirin therapy within one year of DM diagnosis. In contrast, the patients had a seven-year duration (median) of DM in both the ASCEND trial and the JPAD trial.

The incidence of composite cardiovascular events in this study was 15.67 per 1000 person-years in the control group. This was quite similar compared to the report from the Framingham Heart Study [15]. In this report, the incidence of cardiovascular events was 14.7 per 1000 person-years in participants with DM between 1977 and 1995. Therefore, the risk of cardiovascular disease in our study is comparable to the large epidemiology study. Under this premise, our study found no effectiveness of aspirin in primary prevention.

We further analyzed the relationship between the number of CVD risk factors (other than DM) and the effectiveness of aspirin in subgroup analysis (Table 3). There were several well-known CVD risk factors in patients with diabetes, including hypertension, elevated low-density lipoprotein, smoking, and microalbuminuria. Because of the limitation of the National Health Insurance Research Database of Taiwan (NHIRD), we could only select age, hypertension, hyperlipidemia, and CKD for our analysis. Theoretically, patients who have more risk factors are more likely to benefit from aspirin therapy. However, it was shown that the effectiveness of aspirin was the same across all endpoints, regardless of the number of risk factors. Aspirin did not provide extra benefits in patients with a greater number of risk factors.

In the ASCEND trial, aspirin showed an increase in major bleeding events compared with the placebo (rate ratio: 1.29; 95% CI: 1.09 to 1.52), which was mainly driven by an increase in gastrointestinal bleeding and other major bleeding (mainly hematuria and epistaxis). Exploratory analyses showed that the effect on bleeding was consistent over time [2]. In the JPAD trial, there was no significant difference in the composite of hemorrhagic stroke and gastrointestinal bleeding between two groups [3]. In this study, the hospitalized bleeding event rate was slightly higher in the aspirin group than in the non-aspirin group (aHR:1.09; 95% CI: 1.04 to 1.18), with most of the excess being gastrointestinal bleeding and urinary tract bleeding. The result was consistent with the ASCEND trial. Both the ASCEND trial and this study showed no significant difference in intracranial bleeding between groups. In the ASCEND trial, the net benefits of aspirin were largely counterbalanced by the bleeding hazard. However, in this study, we found that aspirin increased the risks of both composite cardiovascular events and bleeding.

Several meta-analyses were conducted to evaluate the role of aspirin in primary prevention in diabetic patients [6,12,13,16]. One of them included seven trials with 11,618 diabetic patients. Aspirin therapy was not associated with a reduction in major cardiovascular events (relative risk: 0.92; 95% CI: 0.83 to 1.02) and did not increase the risk of major bleeding (relative risk: 2.46; 95% CI: 0.70 to 8.61). It is noteworthy that aspirin therapy could reduce MI in men in meta-regression [13]. These meta-analyses shared mostly the same trials; therefore, they had similar conclusions that aspirin showed no definitive benefits as a primary prevention in diabetic patients.

Despite employing propensity score matching to balance comorbidities between study groups, residual confounding by unmeasured variables, such as smoking, body weight, and abnormal laboratory data (e.g., coagulopathy, low platelet counts, impaired liver/renal function), cannot be excluded. Additionally, selective prescribing behaviors by clinicians—who are likely to prescribe aspirin to patients perceived as having a higher cardiovascular risk and avoid it in those with a higher bleeding risk—may also introduce bias. Such factors are not detectable through ICD codes. Furthermore, if it is indeed the case that the aspirin group had a higher baseline cardiovascular risk, insights from the Kaplan–Meier curves (Figure 2b–f) suggest that aspirin does not reduce cardiovascular risk, as there is no long-term benefit evident in these curves. If aspirin was effective in reducing cardiovascular disease (CVD) risk, one might expect to see the curves diverge over time. However, the relative risk represented by the slope of the curves remains consistent throughout the follow-up period, indicating that aspirin may not effectively reduce the risk of CVD.

## 5. Conclusions

In the largest real-world practice study among Asian patients with newly diagnosed diabetes mellitus, we observed that low-dose aspirin was significantly associated with a lower risk of all-cause mortality. However, low-dose aspirin showed no benefit for composite cardiovascular events and was associated with an increase in hospitalized bleeding. Stratified analysis revealed that in patients with more than one risk factor in addition to diabetes, the number of risk factors did not affect the effectiveness and safety of aspirin. These findings suggest that the role of aspirin as a primary prevention in Asian diabetic patients may need to be reconsidered.

## Figures and Tables

**Table 1:** The baseline characteristics among study groups before or after propensity score matching.

	Before PSM			After PSM		
	Non-Aspirin	Aspirin	ASD	Non-Aspirin	Aspirin	ASD
*N*	83,174	41,587		37,095	37,095	
Year of DM diagnosis			0.000			0.006
2006–2010	58,982 (70.91%)	29,491 (70.91%)		25,989 (70.05%)	26,068 (70.26%)	
2011–2015	24,192 (29.08%)	12,096 (29.08%)		11,106 (29.94%)	11,027 (29.74%)	
Sex			0.000			0.008
Male	47,418 (57.01%)	23,709 (57.01%)		20,879 (56.29%)	21,058 (56.77%)	
Female	35,756 (42.99%)	17,878 (42.99%)		16,216 (43.71%)	16,037 (43.23%)	
Age			0.000			0.032
20–50	18,132 (21.80%)	9066 (21.80%)		7374 (19.88%)	7973 (21.49%)	
50–60	29,486 (35.45%)	14,743 (35.45%)		13,024 (35.11%)	13,101 (35.32%)	
60–70	21,302 (25.61%)	10,651 (25.61%)		9787 (26.38%)	9540 (25.72%)	
≥70	14,254 (17.14%)	7127 (17.14%)		6910 (18.63%)	6481 (17.47%)	
Comorbidity						
Hypertension	40,409 (48.58%)	31,504 (75.75%)	0.584	27,687 (74.64%)	27,085 (73.02%)	0.037
Chronic liver diseases	12176 (14.64%)	5161 (12.41%)	0.065	4750 (12.80%)	4715 (12.71%)	0.003
Malignancy	4225 (5.08%)	1366 (3.28%)	0.090	1300 (3.50%)	1301 (3.51%)	0.000
Hyperlipidemia	35,875 (43.13%)	25,102 (60.36%)	0.350	21,834 (58.86%)	21,463 (57.86%)	0.020
Chronic kidney diseases	3329 (4.00%)	1974 (4.75%)	0.036	1698 (4.58%)	1706 (4.60%)	0.001
Peptic ulcer	2842 (3.42%)	1538 (3.70%)	0.015	1340 (3.61%)	1325 (3.57%)	0.002
Gout	8064 (9.70%)	3034 (7.30%)	0.086	2796 (7.54%)	2826 (7.62%)	0.003
Dementia	6751 (8.12%)	3616 (8.70%)	0.021	3229 (8.70%)	3224 (8.69%)	0.000
Medication						
Biguanides	40,785 (49.04%)	26,750 (64.32%)	0.312	23,135 (62.37%)	23,008 (62.02%)	0.007
Sulfonylureas	31,749 (38.17%)	20,832 (50.09%)	0.242	17,776 (47.92%)	17,782 (47.94%)	0.000
Alpha glucosidase inhibitors	5316 (6.39%)	4396 (10.57%)	0.150	3406 (9.18%)	3467 (9.35%)	0.006
Thiazolidinediones	3426 (4.12%)	3013 (7.25%)	0.135	2290 (6.17%)	2339 (6.31%)	0.005
DPP-4	3383 (4.07%)	2740 (6.59%)	0.112	2183 (5.88%)	2205 (5.94%)	0.003
Insulin	2751 (3.31%)	1523 (3.66%)	0.019	1255 (3.38%)	1285 (3.46%)	0.004
NSAIDs	30,578 (36.76%)	15,817 (38.03%)	0.026	14,103 (38.02%)	14,005 (37.75%)	0.005
Systemic corticosteroids	6433 (7.73%)	3217 (7.74%)	0.000	2836 (7.65%)	2861 (7.71%)	0.003
Statin	22,711 (27.31%)	20,445 (49.16%)	0.462	16,747 (45.15%)	16,808 (45.31%)	0.003
Fibrates	8532 (10.26%)	7743 (18.62%)	0.240	6170 (16.63%)	6126 (16.51%)	0.003
PPI	4487 (5.39%)	1480 (3.56%)	0.089	1372 (3.70%)	1399 (3.77%)	0.004
Alpha-blockers	2345 (2.82%)	1771 (4.26%)	0.078	1403 (3.78%)	1475 (3.98%)	0.010
Beta-blockers	15,320 (18.42%)	13,315 (32.02%)	0.317	10,860 (29.28%)	10,931 (29.47%)	0.004
CCBs	23,856 (28.68%)	19,924 (47.91%)	0.404	17,012 (45.86%)	16,793 (45.27%)	0.012
ACEI/ARBs	25,317 (30.44%)	24,408 (58.69%)	0.404	19,783 (53.33%)	20,243 (54.57%)	0.012
Diuretics	7387 (8.88%)	6775 (16.29%)	0.240	5159 (13.91%)	5386 (14.52%)	0.018

PSM = propensity score matching; ASD = absolute standardized difference; DM = diabetes mellitus; DPP-4 = Dipeptidyl peptidase 4; NSAIDs = non-steroidal anti-inflammatory drugs; PPI = proton pump inhibitor; CCBs = calcium channel blockers; ACEI = angiotensin-converting enzyme inhibitor; ARB = angiotensin receptor blocker.

**Table 2:** The efficiency and safety analysis for low-dose aspirin use in the PSM population.

	Non-Aspirin, *n* = 37,095			Aspirin, *n* = 37,095			Crude RR	Adjusted HR
	Person-Years	Event	Rate †	Person-Years	Event	Rate †		
All-cause mortality	237,501.5	3734	15.72	240,716.7	3487	14.49	0.915 (0.874–0.959)	0.916 (0.874–0.959)
Primary composite endpoint	228,049.3	3574	15.67	226,699.8	4636	20.45	1.306 (1.250–1.364)	1.336 (1.279–1.396)
Non-fatal AMI	236,442.5	459	1.94	239,094.3	616	2.58	1.322 (1.172–1.492)	1.327 (1.175–1.497)
Non-fatal Ischemic stroke	231,537.7	2162	9.34	231,742.1	2907	12.54	1.343 (1.271–1.420)	1.375 (1.300–1.453)
Heart failure	234,600.0	1325	5.65	237,085.8	1553	6.55	1.154 (1.072–1.241)	1.175 (1.092–1.265)
Coronary revascularization	236,174.4	448	1.90	237,802.9	870	3.66	1.928 (1.720–2.160)	1.940 (1.731–2.174)
PCI	236,316.2	402	1.70	238,113.0	770	3.23	1.900 (1.684–2.143)	1.912 (1.695–2.157)
CABG	237,329.6	58	0.24	240,296.9	134	0.56	2.278 (1.674–3.100)	2.268 (1.666–3.087)
All hospitalized bleeding	219,879.0	4130	18.78	223,967.5	4166	18.60	1.085 (1.032–1.140)	1.083 (1.030–1.138)
Gastrointestinal bleeding	228,824.9	2218	9.69	232,925.3	2173	9.33	1.080 (1.011–1.154)	1.083 (1.014–1.157)
Intracranial bleeding	234,135.3	891	3.81	237,847.8	828	3.48	0.945 (0.848–1.053)	0.944 (0.847–1.052)
Hematuria	232,022.4	1139	4.91	234,850.5	1248	5.31	1.160 (1.054–1.278)	1.161 (1.054–1.279)

† per 1000 person-years. Crude relative risk (RR) was estimated using a univariate generalized linear model with the assumption that the response variable has a Poisson distribution. Adjusted hazard ratio (HR) was estimated by competing Cox regression, controlling for covariates, including sex, age, comorbidities, and medications listed in Table 1. Follow-up months: for mortality, median = 78 (control), 79 (exposure), max = 144; for non-fatal CVDs, median = 73, 72, max = 144; for bleeding, median = 73, 74, max = 144. PSM = propensity score matching; RR = relative risk; HR = hazard ratio; AMI = acute myocardial infarction; PCI = percutaneous coronary intervention; CABG = coronary artery bypass graft.

**Table 3:** The effectiveness and safety analysis stratified by the number of risk factors.

	Rate (per 1000 Person Years)		
	Non-Aspirin	Aspirin	aHR
For all-cause mortality			
Number of risk factor ‡			
0	16.08	16.03	0.937 (0.806–1.091)
1	14.57	14.30	0.952 (0.889–1.018)
2	16.79	15.97	0.971 (0.910–1.038)
3	42.95	36.58	0.854 (0.747–0.978)
4	59.22	55.51	0.749 (0.410–1.368)
*p* for interaction			0.5645
Composite cardiovascular events			
Number of risk factors ‡			
0	13.80	24.86	1.715 (1.479–1.988)
1	15.73	23.03	1.471 (1.384–1.564)
2	17.26	24.72	1.509 (1.420–1.604)
3	37.44	51.80	1.459 (1.275–1.670)
4	52.74	69.14	1.713 (0.866–3.386)
*p* for interaction			0.3512
For acute myocardial infarction			
Number of risk factors ‡			
0	2.09	2.41	1.117 (0.745–1.676)
1	1.90	2.56	1.331 (1.118–1.585)
2	2.11	2.56	1.200 (1.007–1.430)
3	4.00	3.82	0.963 (0.628–1.475)
4	5.34	9.84	2.228 (0.49–10.131)
*p* for interaction			0.6049
For ischemic stroke			
Number of risk factors ‡			
0	8.03	18.01	2.153 (1.791–2.587)
1	10.19	16.00	1.583 (1.470–1.705)
2	10.49	15.83	1.587 (1.471–1.713)
3	19.58	32.63	1.698 (1.427–2.020)
4	30.18	37.94	1.344 (0.651–2.774)
*p* for interaction			0.0280
For heart failure			
Number of risk factors ‡			
0	5.06	6.58	1.253 (0.973–1.613)
1	5.18	5.66	1.089 (0.975–1.216)
2	6.42	7.49	1.197 (1.081–1.325)
3	17.72	18.92	1.062 (0.870–1.296)
4	31.03	44.75	1.595 (0.793–3.210)
*p* for interaction			0.5620
For hospitalized bleeding			
Number of risk factors ‡			
0	18.91	18.17	1.072 (0.907–1.267)
1	18.74	18.61	1.056 (0.984–1.134)
2	19.37	20.16	1.144 (1.065–1.230)
3	36.66	33.66	1.049 (0.886–1.244)
4	54.44	68.47	1.384 (0.720–2.660)
*p* for interaction			0.5407

‡ risk factors: including age > 75 years old, hypertension, hyperlipidemia, chronic kidney disease.

## Data Availability

Due to the policy of the National Health Insurance Administration in Taiwan, the raw data of this study are not available.
